# A Randomized Study to Compare the Efficacy Between Intranasal and Intravenous Dexmedetomidine for the Removal of Foreign Bodies in the Esophagus at the Cricopharynx Level in Pediatric Patients

**DOI:** 10.7759/cureus.73909

**Published:** 2024-11-18

**Authors:** Manoj Kumar, Prashant Mishra, Amit K Singh, Rahul Gupta

**Affiliations:** 1 Anaesthesiology, Uttar Pradesh University of Medical Sciences, Etawah, IND

**Keywords:** anesthesia, dexmedetomidine, foreign body ingestion, intranasal dexmedetomidine, intravenous dexmedetomidine, pediatric foreign body ingestion, pharyngeal foreign body

## Abstract

Background: Foreign body (coins, magnets, button batteries, and metallic foreign bodies) ingestion is common and causes significant morbidity and mortality in children aged six months to three years. Endoscopic removal of swallowed foreign substances is widely accepted, but sedation and general anesthesia may be required to alleviate pain and anxiety during the procedure. Dexmedetomidine is used as a sedative, hypnotic, anxiolytic, and analgesic. In this study, we aimed to investigate and compare the efficacy between the intranasal (IN) and intravenous (IV) routes for the administration of dexmedetomidine as an adjunct to propofol for the removal of foreign bodies in the esophagus at the cricopharynx level in pediatric patients.

Materials and method: This prospective, double-blinded, randomized study was conducted on 80 patients of either sex and American Society of Anaesthesiologists physical status (ASA PS) I and II aged one month to 12 years with a foreign body in the esophagus at the cricopharynx level undergoing endoscopic foreign body removal. Patients were randomly allocated into groups A and B, with 40 patients each. In the preoperative area group A, IN, 3 mcg/kg, was dripped equally in both nostrils (supine position). In Group B, IV, 3 mcg/kg, diluted in normal saline, was administered intravenously slowly over 10 minutes. The onset of sedation was assessed using the Ramsay Sedation Scale (RSS) every 10 minutes following dexmedetomidine administration, and when an RSS score of 3 or above was achieved, the patient was transferred to the operation theater. In the operation room, patients were induced with injection propofol in a dose of 2 mg/kg or until the loss of verbal response. Parent separation scale scoring was used to evaluate the child’s behavior on separation from parents as a tool to assess the anxiolytic property of dexmedetomidine. Perioperative pain using the Wong-Baker Faces pain rating scale, heart rate (HR), oxygen saturation (SpO_2_), ECG, and blood pressure (BP) were monitored before drug delivery till the end of the procedure. Statistical analysis was done using SPSS version 24.0 (IBM Corp., Armonk, NY).

Results: The mean time (minutes) in shifting to operation theater (33.8 ± 5) was significantly higher in group A compared to group B (10.7 ± 2.1) (p = 0.001). The mean Ramsay Sedation Score was higher at 10 and 20 minutes in group B as compared to group A. Group B had significantly lower pain (2.2 ± 1.4) (p ≤ 0.001) at 10 minutes, while in group A, the pain score decreased slowly and took more time but remains lower (1.35 ± 0.94) for longer duration (120 minutes) in the postoperative period in comparison to Group B (1.94 ± 0.35) (p = 0.002). Group B had a sharp hemodynamic decrease, while group A had slow, persistent changes.

Conclusion: IN administration of dexmedetomidine is a safer and more effective mode of sedation to remove a foreign body from the esophagus at the cricopharyngeal level in pediatric patients. It provides stable and sustained hemodynamic parameters and longer postoperative pain relief compared to IV dexmedetomidine.

## Introduction

Foreign body ingestion is a frequent condition associated with a high risk of morbidity and mortality. Children are at high risk of foreign body ingestion [1]. The most commonly ingested foreign bodies in children include coins, magnets, button batteries, and metallic objects (parts of toys).

Most swallowed foreign bodies (>90%) will harmlessly pass through the gastrointestinal (GI) tract [2], but some (10%) will lead to health problems if they become lodged in the GI tract [3] or traumatize the mucosa of the GI tract. In these cases, endoscopic removal is required. To assist with the endoscopy, the patient may sometimes need sedation or general anesthesia. Nowadays, dexmedetomidine has been used for sedation and other purposes in pediatric and adult patients.

Dexmedetomidine is a highly selective agonist of the alpha-2 adrenergic receptor, primarily targeting the alpha-2 receptor in the spinal cord and the locus coeruleus nucleus [4]. Dexmedetomidine's unique sedative effect allows for a smooth transition from sleep to waking up, facilitating patient cooperation and communication when stimulated, and it exerts minimal impact on hemodynamics and has a short half-life. Dexmedetomidine exhibits a respiratory-sparing effect, even at higher dosages [5]. It is well-established that intravenous (IV) dexmedetomidine is used for sedation. In addition, it can be injected via the intramuscular, intranasal (IN), and buccal routes. The IN administration of dexmedetomidine has advantages due to its non-invasive nature and reduced anxiety for pediatric patients because cannulation can be difficult and distressing for children.

Some studies are available for IN dexmedetomidine administration, but only a few have compared IN dexmedetomidine with IV dexmedetomidine [6]. The objective of this study was to compare the efficacy of both the IN and IV routes for the administration of dexmedetomidine as an adjunct to propofol for the removal of foreign bodies in the esophagus at the cricopharynx level in pediatric patients.

## Materials and methods

This double-blind, randomized controlled study was approved by our university’s ethics and scientific committee (EC Number: 45/2022-23 dated 22/12/2022). The study was registered at the Clinical Trial Registry of India with Reg. No. CTRI/2023/10/058515 and conducted prospectively from October 2023 to September 2024. The study is in accordance with the Declaration of Helsinki 1964, as revised in 2013, concerning human and animal rights. Written and informed consent was obtained from the patient’s parents before the procedure.

Inclusion and exclusion criteria

This study included pediatric patients classified as American Society of Anaesthesiologists physical status (ASA PS) grades I and II, aged one month to 12 years, who had a foreign body in the esophagus at the cricopharynx level and were undergoing endoscopic foreign body removal. The study excluded patients with an ASA score of III and IV, parental refusal, foreign body in the upper or lower respiratory tract, known allergy or hypersensitivity to dexmedetomidine, organ dysfunction, mental retardation, cardiac arrhythmia, congenital structural anomalies or disorders, severe preoperative respiratory impairment, and a family history of malignant hyperthermia. The study also excluded patients who required an additional dose of dexmedetomidine or ketamine.

Sample size calculation and randomization

The sample size was calculated by using the following formula:  ​​​​​​

$n=[Z_{1-\alpha/2}\sqrt{\left( r+1 \right)p\left( 1-p \right)}+Z_{1-\beta}\sqrt{rp_{1}\left( 1-p_{1} \right)+p_{2}\left( 1-p_{2} \right)}]^{2}/r\left( p_{1}-p_{2} \right)^{2}$​​

Here, n = sample size, Z_1−α/2_ = 1.96 at a 95% confidence level (α = 0.05), Z_1−β_ = 0.84 at an 80% power of the study (β = 0.2), r = ratio (group 2/group 1, taken as 1), p = average value of p_1_ and p_2_, p_1_= prevalence of sedation in the IN group in the previous study, p_2_ = prevalence of sedation in the IV group in the previous research, (p_1_−p_2_) = difference in the prevalence of sedation in the IN and IV groups. In previous studies, p_1_ = 0.91 [7] and p_2_ = 0.67 [8]. On applying the formula, n = 39. Hence, the final sample size was taken as 40 for each group. A total of 80 patients of either sex of the age one month to 12 years were taken and randomly divided into two groups.

A random number table for 80 patients was prepared, and using sequentially numbered opaque sealed envelopes, the patients were randomly assigned into two groups. Group A (n = 40) patients received IN dexmedetomidine (3 mcg/kg), and group B (n = 40) patients received IV dexmedetomidine (3 mcg/kg). To ensure double blinding, the group A patients were given IV normal saline infusion, and in group B patients, IN normal saline was dripped in both nostrils. Moreover, the anesthesiologist who administered the study drug was not involved in data collection, and the observer who took all the vitals recording and score assessment was also unaware of the group allocation.

Procedure

A pre-anesthetic check-up, including the patient’s detailed history, general, systemic examination, and clinical laboratory tests, was conducted. The parents of the patients were explained about the concerned technique, and written and informed consent was obtained from them. Prior to the procedure, all patients were advised to fast for six hours for solid food and two hours for clear fluid. IV cannula access had been established in wards for all the children before shifting to the preoperative area. Patients along with their parents were brought to the preoperative area, and the patient’s nil per oral status and consent were checked. Routine non-invasive monitors were attached, and baseline (five minutes prior to drug administration) readings of the heart rate (HR), non-invasive blood pressure (NIBP), and peripheral oxygen saturation (SpO_2_) were recorded. Ringer lactate infusion was started at 5-10 ml/kg. For the study, dexmedetomidine with a concentration of 100 mcg/ml was used in both groups. In group A, all patients were kept supine. For IN administration, dexmedetomidine was diluted according to the desired dose of 3 mcg/kg of body weight to make the final volume of 2 ml. Diluted dexmedetomidine was dripped into each nostril using a syringe. After drug administration, small children were kept in the same position for two to three minutes with the help of parents while gently massaging the alae of the nose to facilitate drug absorption by the nasal mucosa. In group B, a single dose of dexmedetomidine at 3 mcg per kg [9] diluted in 0.9% saline was administered via slow IV infusion over a duration of 10 minutes in the preoperative area. HR, SpO_2_, and NIBP were measured before and every five minutes after drug administration until the completion of the procedure. The onset of sedation was evaluated by the Ramsay sedation scale [10] preoperatively at every 10 minutes after the administration of dexmedetomidine (Table 1).

**Table 1:** Ramsay sedation score

Patient status	Score
1. The patient is anxious, agitated, and impatient.	1
2. The patient is cooperative, oriented, and calm.	2
3. The patient only responds to verbal commands.	3
4. The patient demonstrates a brisk response to the glabellar tap test or auditory stimulus.	4
5. The patient demonstrates a sluggish response to the glabellar tap test or auditory stimulus.	5
6. The patient does not respond to the glabellar tap test or auditory stimulus.	6

A Ramsay sedation score of 3 or more was considered satisfactory for shifting the patient into the operation theater. When a child was taken away from their parents to the operation theater, a four-point parent separation scale [11] scoring was used to evaluate the child’s behavior on separation from parents (Table 2).

**Table 2:** Parent separation score

Score	Grade	Patient’s behavior
1	Excellent	The patient is unafraid, cooperative, asleep, and separate easily.
2	Good	Slight fear or crying, easy to calm, quiet with reassurance
3	Fair	Moderate fear, cries, no clinging, not calm with reassurance
4	Poor	Crying, clinging to their parent, need for restraint

If the child showed extreme resistance, 1 mg/kg of ketamine was injected intravenously, and that patient was excluded from the study.

In the operation theater, after applying the standard monitors, HR, NIBP, electrocardiography (ECG), and SpO_2_ monitoring were carried out throughout the intraoperative and postoperative period. Nasal prongs were applied to each patient. All the patients were pre-medicated with an IV injection glycopyrrolate (5 mcg/kg), injection fentanyl in a dose of 2 mcg/kg with injection midazolam (0.05 mg/kg). After three minutes of preoxygenation, anesthesia was induced (intraoperative time 0 minutes) with IV injection propofol (2 mg/kg) or in a dose sufficient for the loss of verbal response. After induction, the patient was handed over to the ENT surgeon for removal of the foreign body using a rigid esophagoscope. During this time, apneic oxygenation was provided to the patient with nasal prongs at a rate of 15 liters per minute to prolong the apnea time and allow more time for the surgeon to perform the procedure before desaturation [12]. If the patient’s SpO_2_ level fell below 90%, oxygenation was carried out with 100% oxygen via bag and mask ventilation, and again, 100% SpO_2_ was achieved before handing over the patient to the surgeon once more.

The preoperative duration (from the administration of the drug until shifting the patient into the operation theater) and the intraoperative duration (duration of the procedure) were meticulously recorded. If the duration of the procedure exceeded more than one hour or the patient needed intubation, those patients were excluded from the study. If required, a rescue dose of 1 mcg/kg of dexmedetomidine was given, and those patients were also excluded from the study. Heart rate, oxygen saturation, and non-invasive blood pressure monitoring were continued during the procedure.

Pre- and intraoperative pain assessment was conducted at intervals of every 10 minutes and hourly after 60 minutes during the postoperative period using the Wong-Baker Faces Pain Rating Scale, where 0 = no pain, 2 = a little pain, 4 = a little more pain, 6= even more pain, 8 = a whole lot of pain, and 10 = worst pain. The assessment continued until the Wong-Baker Faces Pain Rating Scale score reached 2 or higher, and the corresponding time was carefully recorded.

The primary objectives were to assess the onset and level of sedation, perioperative pain, and hemodynamic variables between the IN and IV routes for dexmedetomidine administration in pediatric patients preoperatively. The secondary objectives included evaluating anxiety levels during parental separation, measuring the procedure's duration, and monitoring for side effects or complications, such as oxygen desaturation, episode of bradycardia, hypotension, coughing, body movements, bronchospasm, laryngospasm, and breath holding during the procedure and in the postoperative phase.

Statistical analysis

The analysis included profiling of patients on different demographic, clinical, and hemodynamic parameters. The Shapiro-Wilk test was used to test the normality assumption of quantitative data. An independent student t-test was used to test the mean between independent groups. Cross tables were generated, and the Chi-square test was used for testing associations. P-value < 0.05 was considered statistically significant in this study. All analyses were done using SPSS software, version 24.0 (IBM Corp., Armonk, NY).

## Results

**Figure 1:**
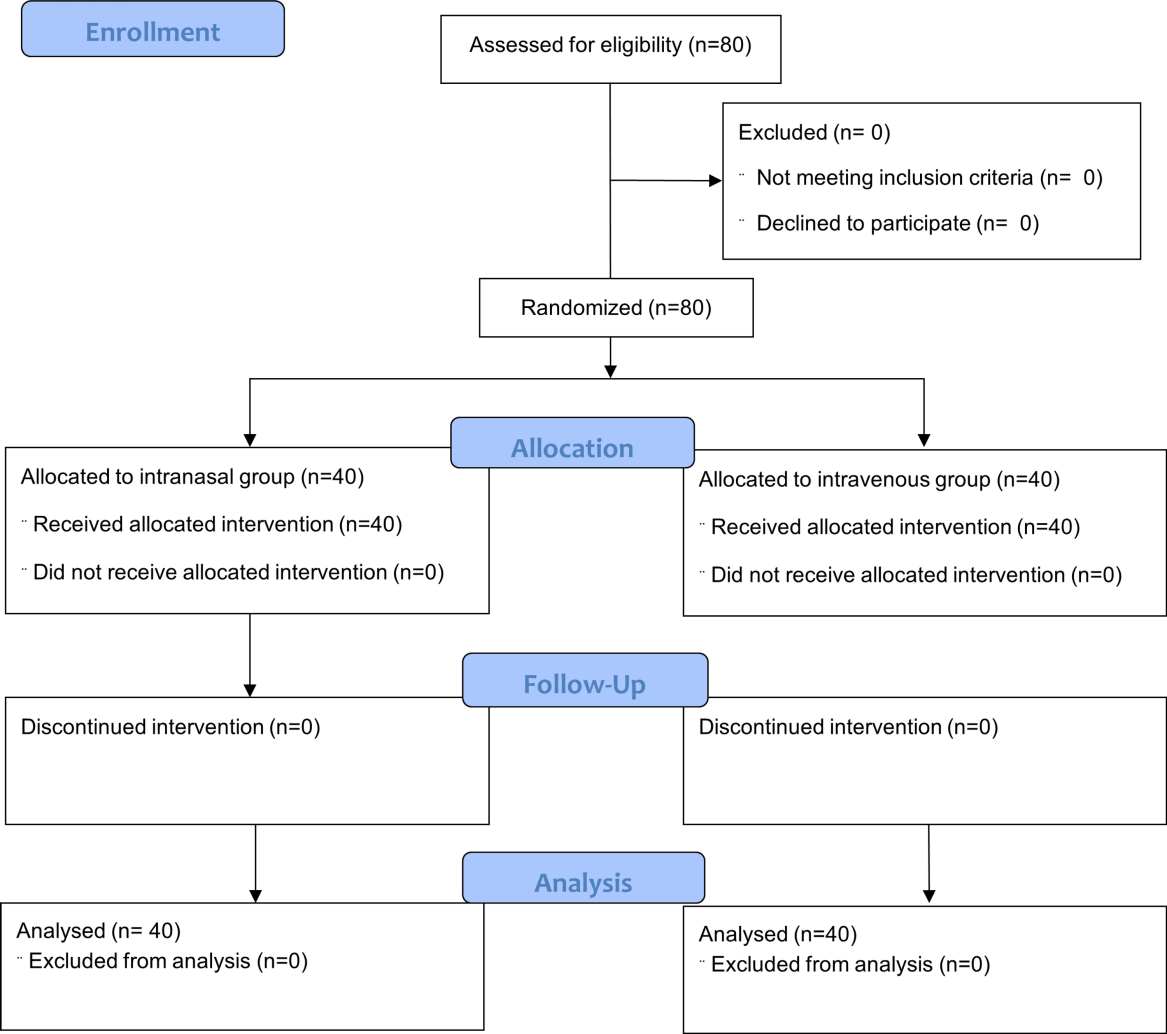
Consolidated Standards of Reporting Trials (CONSORT) flow diagram.

No patients were excluded from the study; 80 patients were randomly divided into two groups of 40 patients each (Figure 1). In our study, the demographic characteristics (age, sex, BMI, and ASA-PS grade), duration of procedure, and parent separation score of patients in groups A and B were shown to be statistically insignificant (p > 0.05) (Table 3).

**Table 3:** Demographic characteristics among the studied groups n: number of patients, ASA PS: American Society of Anaesthesiologists physical status classification, BMI: body mass index, Min: minutes Data expressed in numbers were analyzed by the Chi-square test. Data are expressed in mean, and standard deviation is analyzed by unpaired t-test. P-value < 0.05 is statistically significant.

Parameters	Group A (n = 40)	Group B (n = 40)	P-value	t-value	Chi-square value
Age (years)	5.1 ± 1.9	5.7 ± 2.1	0.189	-1.325	-
Sex (male/female)	16/24	18/22	0.651	-	0.204
BMI (Kg/m^2^)	19.20 ± 2.34	19.25 ± 2.01	0.919	-0.264	-
ASA PS I/II	35/5	33/7	0.531	-	0.392
Parent separation score	1.23 ± 0.42	1.35 ± 0.48	0.222	-1.231	-
Duration of procedure (minutes)	16.7 ± 4.4	17.4 ± 5.6	0.565	-0.577	-

**Figure 2:**
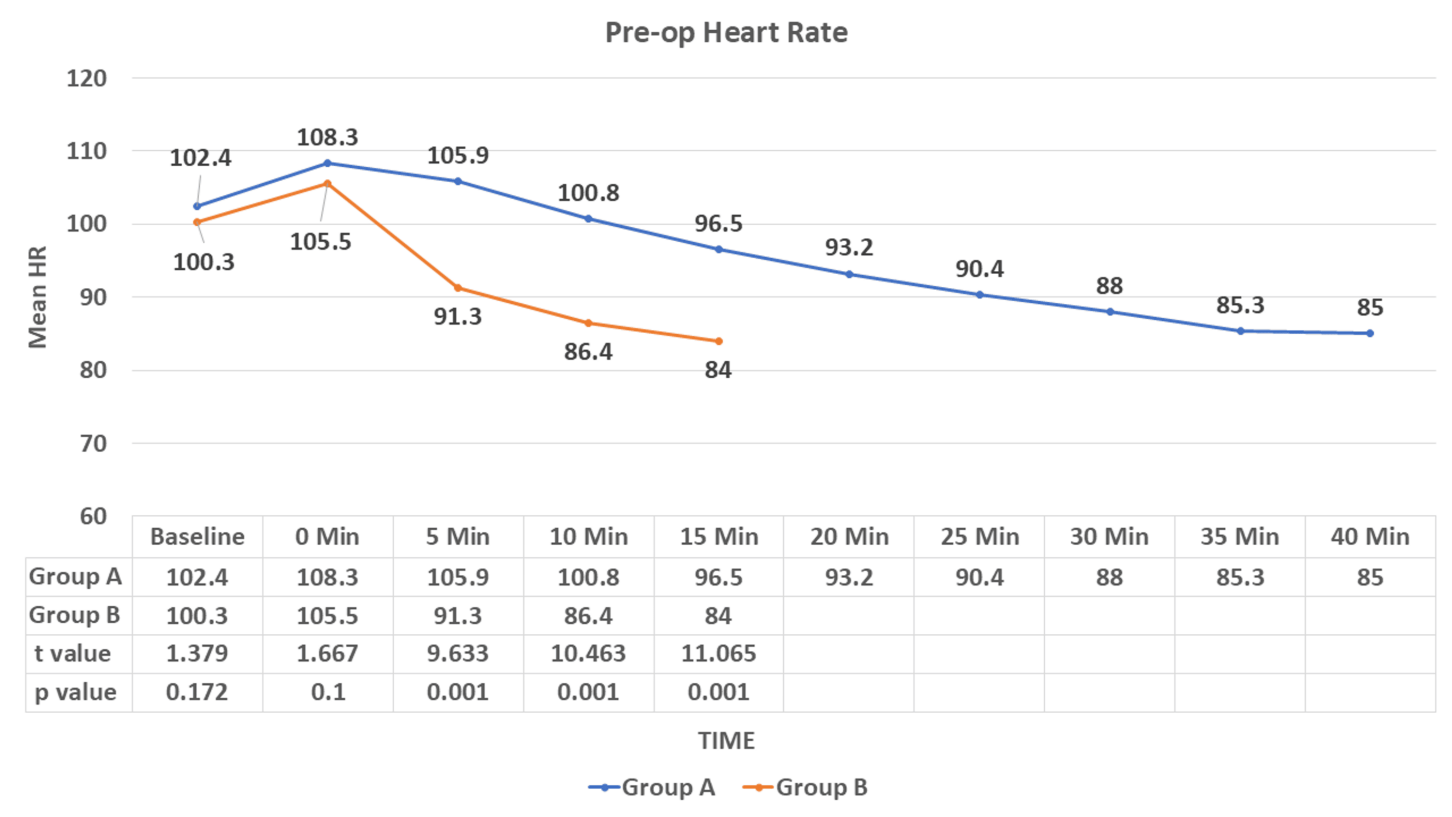
Comparison of the mean heart rate in the preoperative duration between the study groups HR: heart rate Data are expressed as mean ± SD and analyzed by unpaired t-test.

No significant difference was observed in the mean HR at baseline and at 0 minutes (at the time of drug administration) between the study groups in the preoperative area. There was a statistically significant difference in the mean HR after five minutes in the preoperative area between the two groups (p < 0.05). HR was lower in group B than in group A (Figure 2).

**Figure 3:**
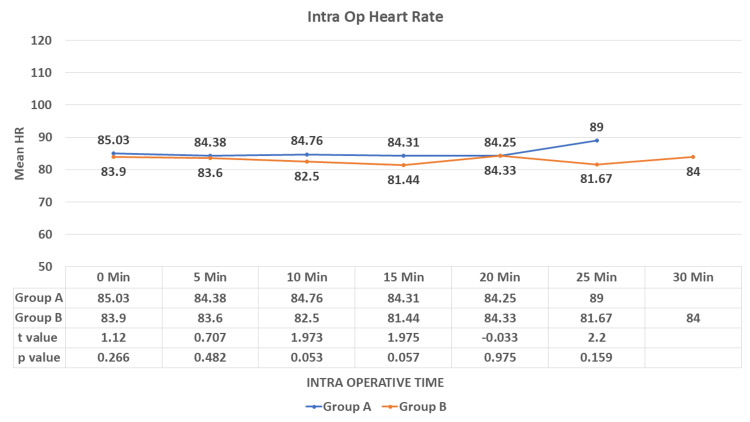
Comparison of the mean heart rate in the intraoperative duration between the study groups. HR: heart rate, Intra op: intraoperative Data are expressed as mean ± SD and analyzed by unpaired t-test.

During intra-operative monitoring, there was no significant difference observed in mean HR between the study groups (p > 0.05) (Figure 3).

**Figure 4:**
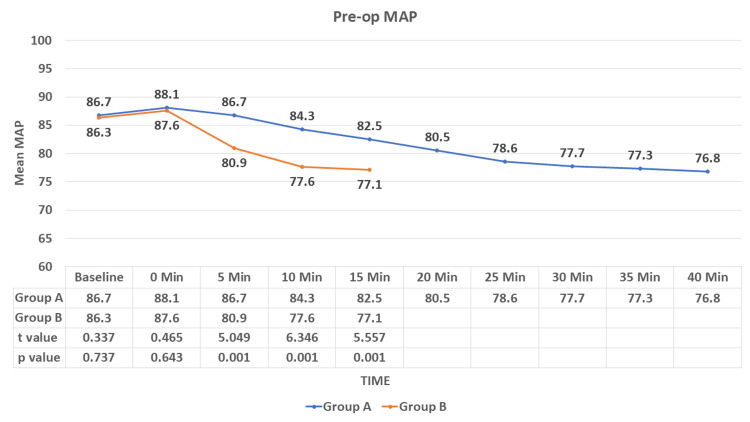
Comparison of the mean arterial pressure (pre-op) among the groups MAP: mean arterial pressure, Pre op: preoperative Data are expressed as mean ± SD and analyzed by unpaired t-test.

There was no significant difference observed in the mean MAP at baseline (MAP: group A vs. B: 86.7 ± 4.8 vs. 86.3 ± 4.7; p = 0.737) and at 0 minute (MAP: group A vs. B: 88.1 ± 4.8 vs. 87.6 ± 5; p = 0.643) between the groups. From five minutes in the pre-op, there was a significant difference in mean MAP in the two groups (p < 0.05). The mean MAP was low in group B in comparison to group A (Figure 4).

**Figure 5:**
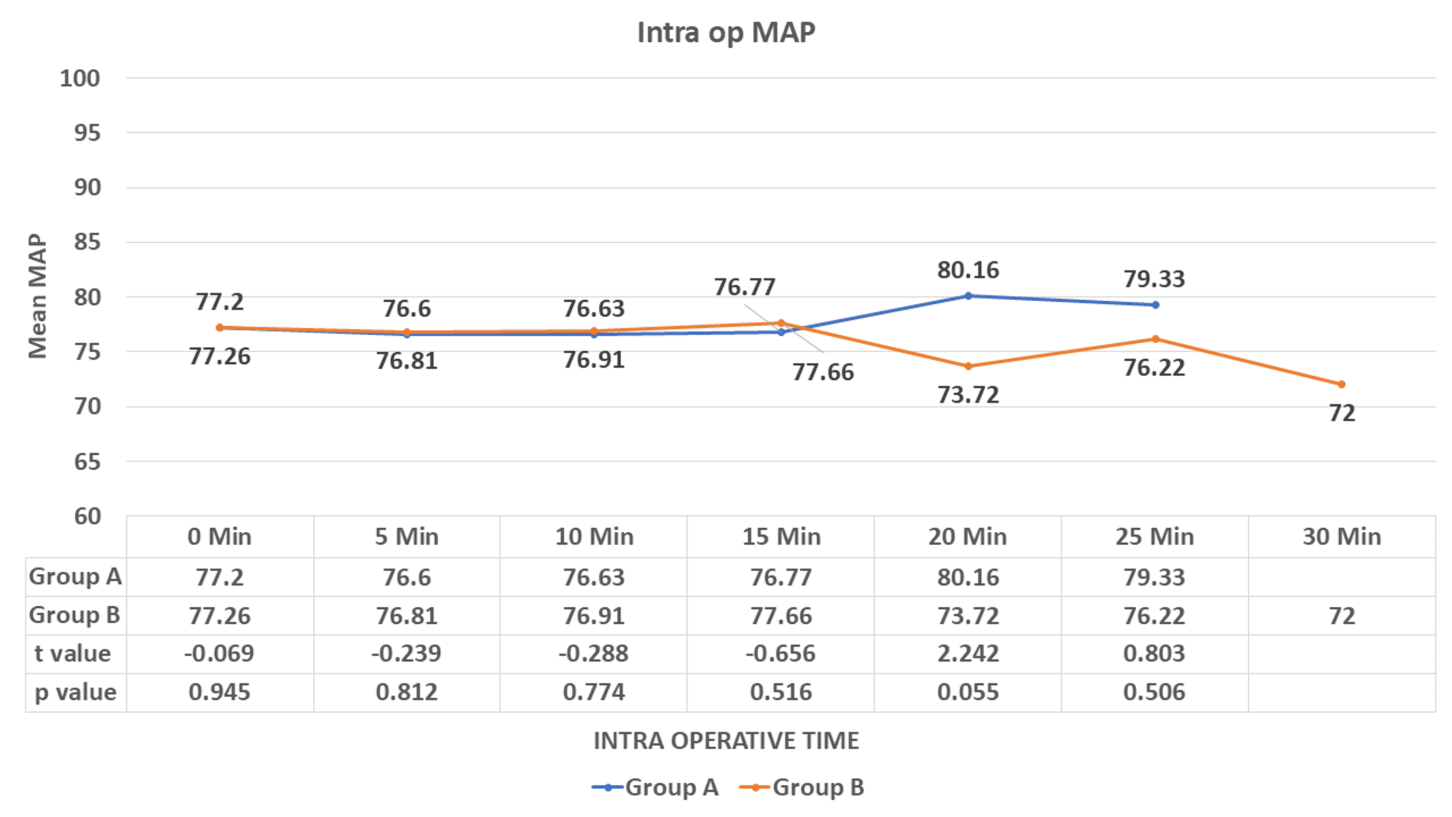
Comparison of the mean arterial pressure (intra-op) among the groups. MAP: mean arterial pressure Data are expressed as mean ± SD and analyzed by unpaired t-test.

There was no significant difference observed in the mean MAP during the procedure between the study groups (p > 0.05) (Figure 5).

**Figure 6:**
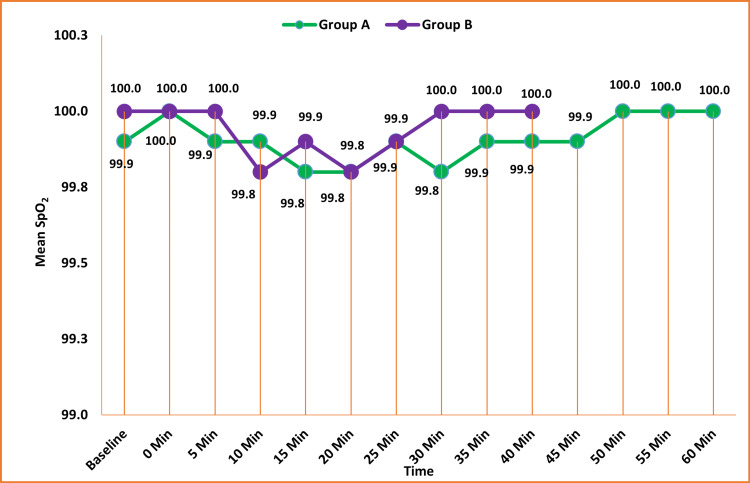
Comparison of the peripheral saturation of oxygen SpO2 (%) among the groups Data are expressed as mean ± SD and analyzed by unpaired t-test.

No significant difference was observed in SpO_2_ from baseline to the last follow-up between the groups (p > 0.05) (Figure 6).

**Figure 7:**
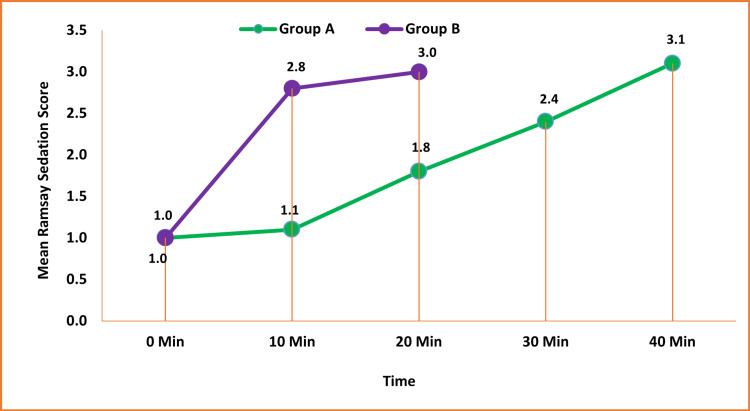
Comparison of the mean Ramsay sedation score between the study groups Data are expressed as mean ± SD and analyzed by unpaired t-test.

The mean Ramsay sedation score was higher at 10 and 20 minutes in group B compared to group A and was statistically significant (p < 0.05) (Figure 7).

**Figure 8:**
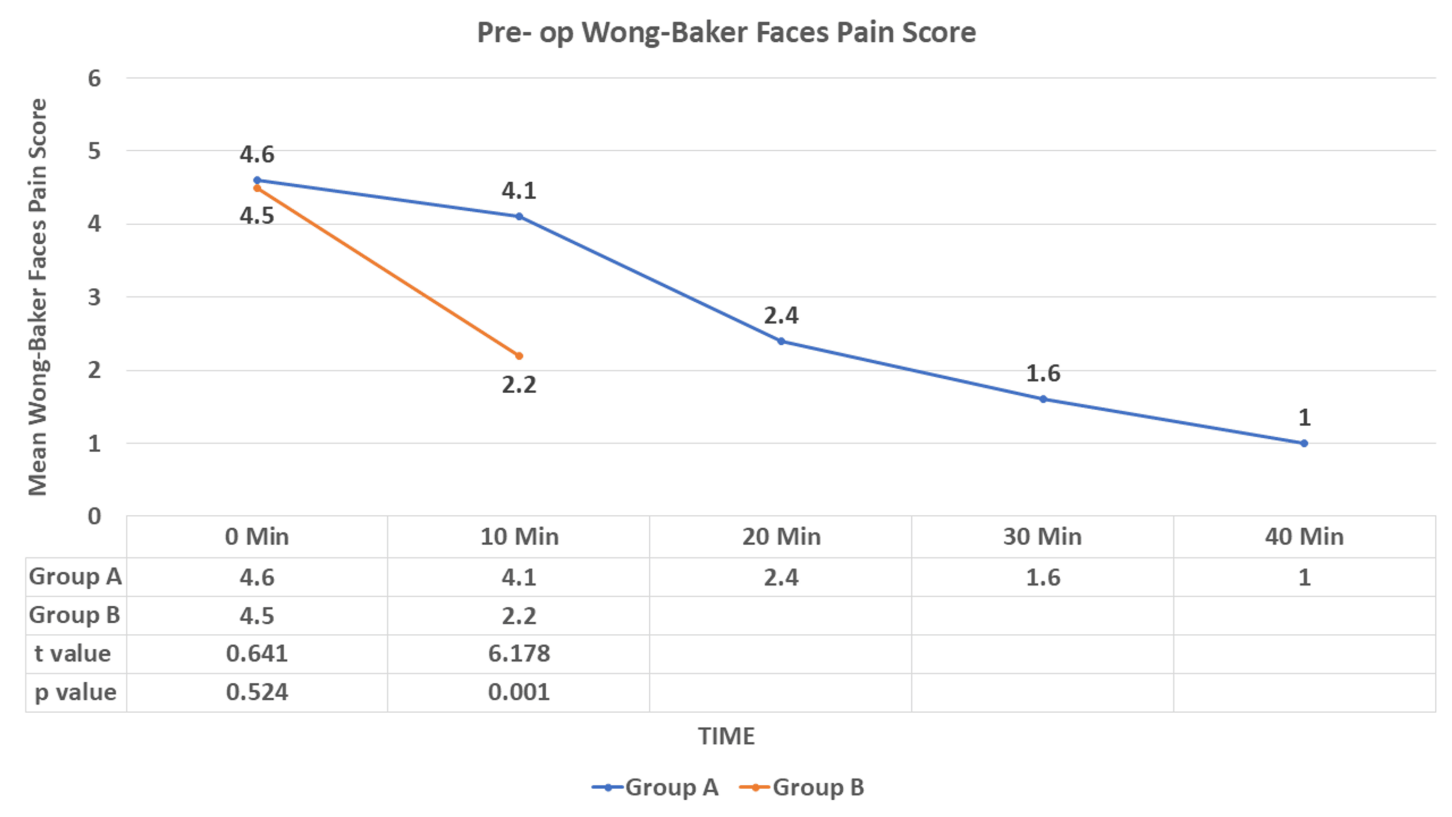
Comparison of the mean Wong-Baker Faces Pain Score in the pre-op between the study groups Pre op: preoperative Data are expressed as mean ± SD and analyzed by unpaired t-test.

In the pre-op, the mean Wong-Baker Faces Pain Score was lower at 10 minutes in group B compared to group A and was statistically significant (p < 0.001) (Figure 8).

**Figure 9:**
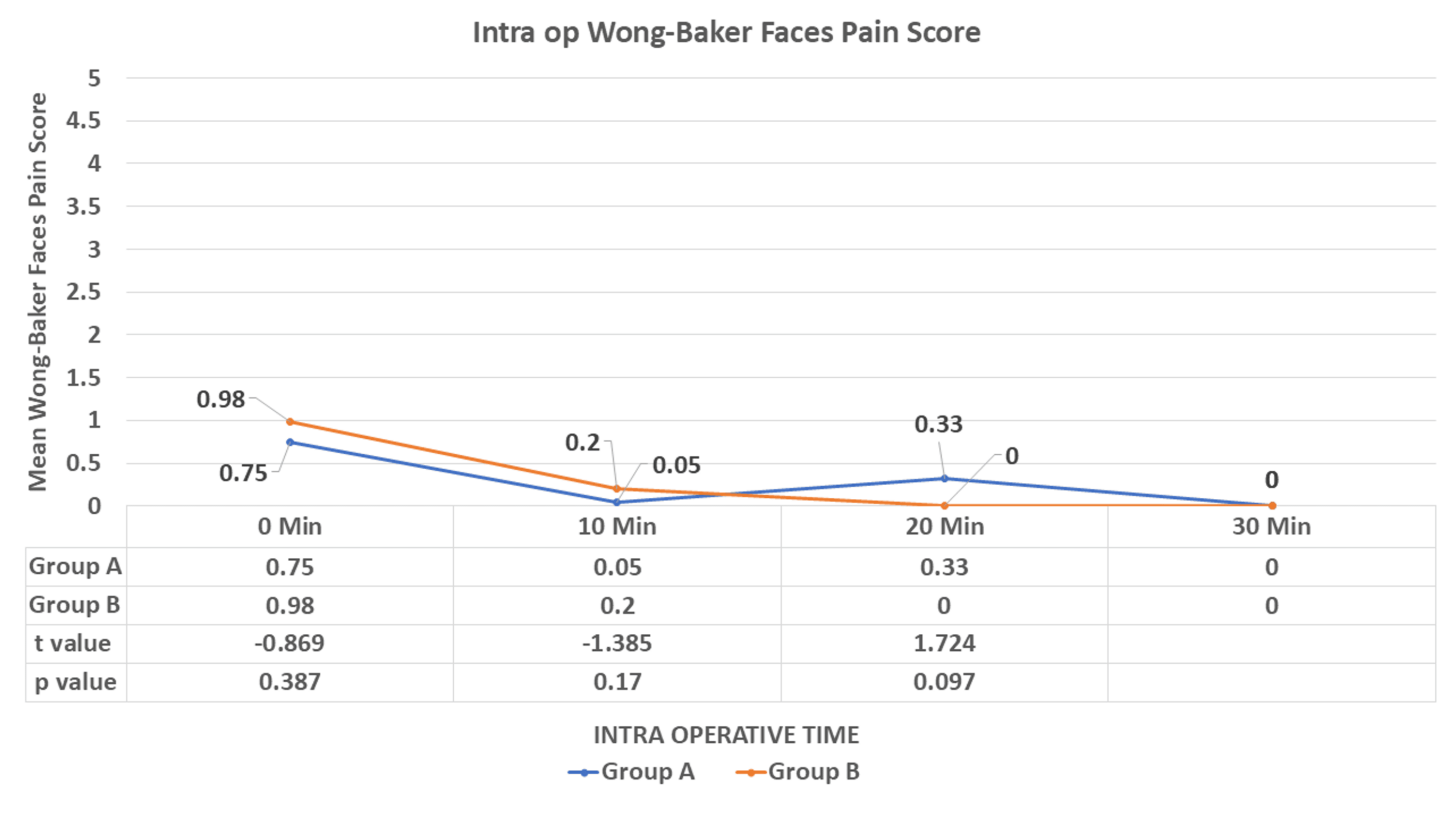
Comparison of the mean Wong-Baker Faces Pain Score during the procedure between the study groups Intra op: intraoperative Data are expressed as mean ± SD and analyzed by unpaired t-test.

After induction (0 minutes) during the intraoperative period, there was no significant difference observed between the study groups (p > 0.05) (Figure 9).

**Figure 10:**
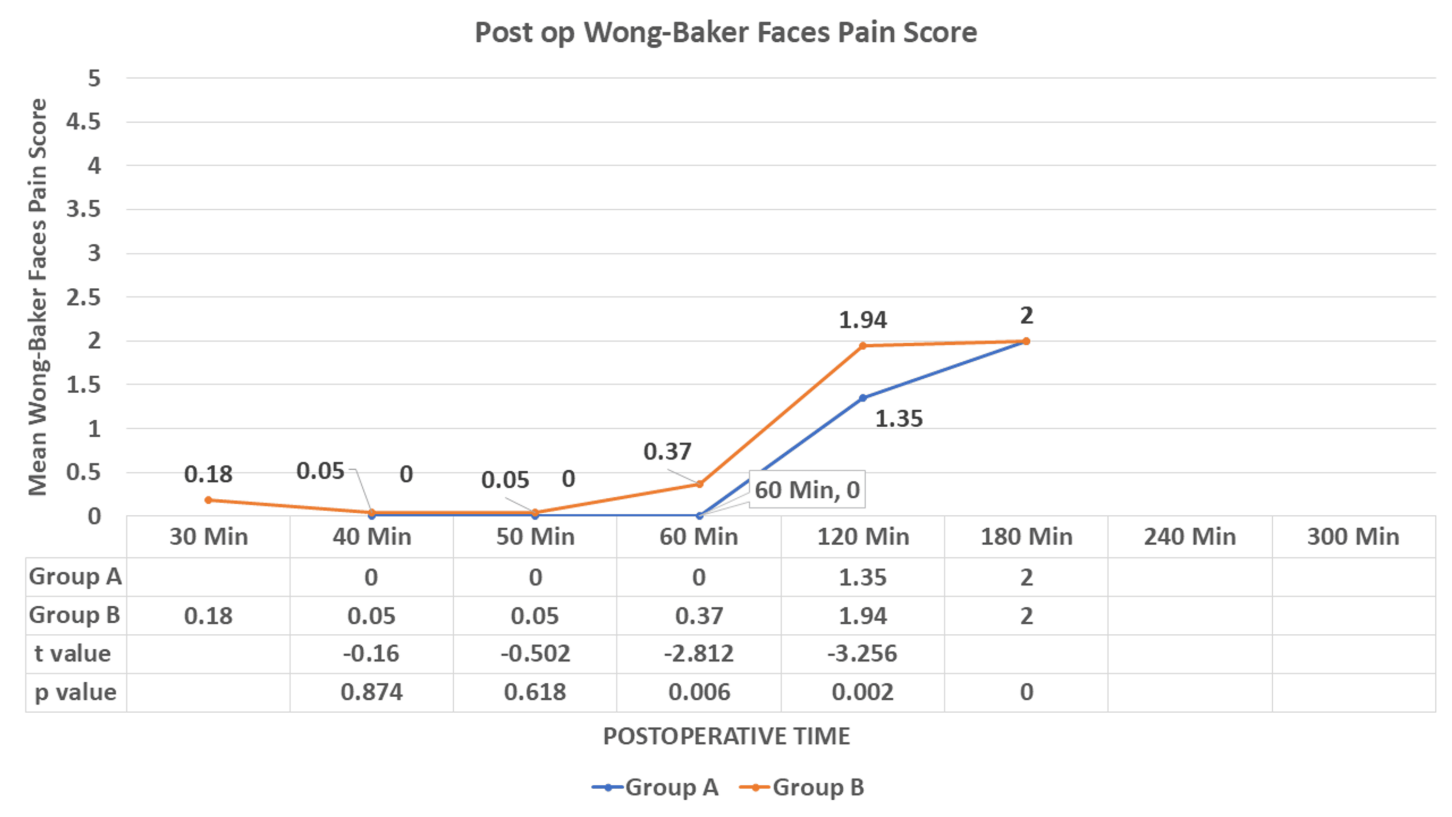
Comparison of the mean Wong-Baker Faces Pain Score after the procedure between the study groups Post op: postoperative Data are expressed as mean ± SD and analyzed by unpaired t-test.

The Wong-Baker Faces Pain Score was higher in group B as compared to group A at 60 and 120 minutes, and the difference was statistically significant (p < 0.05) (Figure 10).

The mean time in shifting patients (minutes) was higher in group A (33.8 ± 5) as compared to group B (10.7 ± 2.1). This difference was statistically significant (p = 0.001) (Table 4).

**Table 4:** Comparison of the mean time in shifting patients from the preoperative room to the operation theater (minutes) between the study groups n: number of patients, SD: standard deviation, SE: standard error, CI: confidence interval Data are expressed as mean ± SD and analyzed by unpaired t-test. P-value < 0.05 statistically significant.

Parameter	Group A (n = 40)	Group B (n = 40)	Mean difference ± SE	95% CI of the difference		t-value	p-value
	Mean ± SD	Mean ± SD		Lower	Upper		
Time in shifting patients (minutes)	33.8 ± 5	10.7 ± 2.1	23.1 ± 0.9	21.4	24.8	27.157	< 0.001*

No adverse events were recorded during the procedure, such as oxygen desaturation, coughing, body movements, bronchospasm, laryngospasm, breath holding, or coughing in the post-anesthesia care unit in either group.

## Discussion

Infants and toddlers explore their world by putting objects in their mouths, placing themselves at risk of having foreign bodies in the esophagus or respiratory tract. For the endoscopic extraction of a foreign body routinely, sedative agents are used, including midazolam, propofol, ketamine, and opioids like fentanyl and meperidine, which may be administered individually or in combination. Nowadays, dexmedetomidine is increasingly used as an adjuvant during endoscopic procedures. Dexmedetomidine has been administered via a variety of routes, but IN is the most preferred route after IV due to its non-invasiveness and ease of administration [13].

In our study, the demographic characteristics, ASA physical status, duration of procedure, parent separation score, and SpO_2_ were comparable with respect to each other (p > 0.05). For this study, the time to shift the patient in the operation theater from the preoperative area is considered as the onset of sedation. The mean time in shifting patients was significantly higher in group A (33.8 ± 5) minutes as compared to group B (10.7 ± 2.1 minutes, p = 0.001). This is because peak plasma levels are reached sooner in IV dosing than in the IN treatment. Our observation was consistent with the study done by Bhargavi M et al. [14], who compared sedation depth with the Ramsay sedation score on 25 anxious patients who had undergone surgical removal of impacted mandibular third molars and in their study, they found that the sedative effect began to take effect between 30 and 45 minutes later after IN administration of dexmedetomidine for sedation. Another study by Behrle et al. [15] used IN dexmedetomidine at 3 mcg/kg, alone or in combination with IN midazolam, for sedation 40 minutes before the procedure and found a 92% success rate. Similar observations were also reported in our study. Ashraf et al. [16] compared propofol and dexmedetomidine during pediatric gastrointestinal endoscopy. Dexmedetomidine 2.5 mcg/kg over 10 minutes with 2 mcg/kg/h infusion resulted in a mean sedation time of 10.51 ± 1.75 minutes, similar to our IV group.

The mean Ramsay sedation score in this study was found to be higher at 10 minutes in group B (2.8 ± 0.5) as compared to group A (1.1 ± 0.3) (p < 0.001) and at 20 minutes in group B (3.0 ± 0.0) as compared to group A (1.8 ± 0.4) (p < 0.001). We observed that the higher Ramsay sedation score was achieved earlier in the IV dexmedetomidine group compared to the IN group. Ambi et al. [17] gave IN dexmedetomidine 2 mcg/kg 30 minutes before the MRI scan, resulting in mean sedation scores (University of Michigan Sedation Scale) of 1.17 ± 1.6 and 2.60 ± 0.9 after 15 and 30 minutes, respectively, similar outcomes as our study. Janakibabu et al. [18] found a satisfactory sedation status in patients receiving 1 mcg/kg and 2 mcg/kg dexmedetomidine in a prospective double-blinded comparison study of 90 children. The results were similar when assessing the sedation status at the time of parenteral separation and at the time of induction.

In this study, the mean Wong-Baker Faces pain score was lower at 10 minutes (pre-op) in group B as compared to group A (p < 0.001), and at 60 and 120 minutes (post-op), the mean pain score was slightly higher in group B as compared to group A (p = 0.002). Kaye AD et al. [19] stated that the onset of action after an IV dose is usually five to 10 minutes and its peak effect in 15 to 30 min. The IN route has an onset of action in 45 minutes with peak effect in 90 to 100 minutes because, after IV administration, dexmedetomidine undergoes rapid distribution with distribution half time (T1/2) of six minutes, followed by a terminal half-time (T1/2) of two hours. We found similar results in our study in the view of perioperative pain management.

During the study, we observed a decrease in HR in both study groups but a sudden fall in the IV group compared to the IN group, which was statistically significant after five minutes of drug administration (p < 0.001). After induction (intra-op), no significant difference was observed in the mean HR until the end of the study. There was a decrease in SBP, DBP, and MAP in both groups, with a rapid fall in the IV group compared to the IN group. This difference was statistically significant in the pre-op after five minutes of drug administration (p < 0.001). No significant difference was observed in SBP, DBP, and MAP in the intra-op and till the end of the procedure. Munro et al. [20] reported a reduction of blood pressure and heart rate by <20% of baseline as a loading dose of 1 mcg/kg dexmedetomidine was administered over 10 minutes followed by an initial infusion rate of 1 mcg/kg/hr. Bhargavi et al. [14] also found a significant difference in the mean HR and SBP readings in their study on 25 patients of surgical removal of impacted mandibular third molars after giving IN and IV dexmedetomidine for sedation. Mason et al. [21] found that IV dexmedetomidine sedation was associated with modest ﬂuctuations in HR and arterial blood pressure, required no pharmacologic interventions, and did not result in any adverse event. These results were consistent with our study.

In our study, the mean parent separation scores in groups A and B were 1.2 ± 0.4 and 1.4 ± 0.5, respectively. No significant difference was observed in the mean parent separation score at the separation between the groups (p = 0.222). Li et al. [22] found a statistically significant difference between dexmedetomidine and normal saline groups in behavior at separation from parents (P = 0.009) but no significant difference between two dexmedetomidine groups. They concluded that IN dexmedetomidine 40 minutes before separation made children less distressed.

Limitations

This study has certain limitations. The study is confined to our institution and exclusively involves pediatric patients undergoing foreign body removal from the esophagus at the cricopharynx level. This limitation reduces the diversity of the patient population available for research. Therefore, a multi-center study incorporating various procedures is essential to understand better the effects and differences associated with different delivery routes. A further limitation of the study was the administration of propofol in both groups, which may have affected hemodynamic parameters. The lack of comparison regarding the dose of propofol administered in both groups represents an additional limitation of this study. Further research should be conducted at lower dosages or without the use of propofol.

## Conclusions

From the observation and analysis of the present study, it was concluded that IN administration of dexmedetomidine as an adjunct to propofol has a slow onset of sedation and an effective mode of analgesia and sedation to remove a foreign body from the esophagus at the cricopharyngeal level in pediatric patients. IN dexmedetomidine provides stable and sustained hemodynamic parameters and longer postoperative pain relief compared to IV dexmedetomidine, so IN dexmedetomidine administration is safer in comparison to IV dexmedetomidine.
